# Ten‐year attrition and antiretroviral therapy response among HIV‐positive adults: a sex‐based cohort analysis from eight West African countries

**DOI:** 10.1002/jia2.25723

**Published:** 2021-05-21

**Authors:** Thierry Tiendrebeogo, Eugène Messou, Shino Arikawa, Didier K Ekouevi, Aristophane Tanon, Vivian Kwaghe, Eric Balestre, Marcel Djimon Zannou, Armel Poda, François Dabis, Antoine Jaquet, Albert Minga, Renaud Becquet, IeDEA West Africa Collaboration

**Affiliations:** ^1^ University of Bordeaux Inserm French National Research Institute for Sustainable Development (IRD) Bordeaux Population Health Research Center Team IDLIC UMR 1219 Bordeaux France; ^2^ Centre de Prise en charge de Recherche et de Formation (Aconda‐CePReF) Abidjan Côte d'Ivoire; ^3^ Département des Sciences Fondamentales et Santé Publique Université de Lomé Lomé Togo; ^4^ Service de Maladies Infectieuses et Tropicales (SMIT) Treichville Teaching Hospital Abidjan Côte d'Ivoire; ^5^ University of Abuja Teaching Hospital Abuja Nigeria; ^6^ Centre de Traitement Ambulatoire (CTA) Centre National Hospitalier Universitaire (CNHU) Cotonou Benin; ^7^ Institut Supérieur des Sciences de la santé Université Polytechnique de Bobo‐Dioulasso Bobo‐Dioulasso Burkina Faso; ^8^ Centre Médical de Suivi des Donneurs de Sang (CMSDS) Centre National de Transfusion Sanguine (CNTS) Abidjan Côte d'Ivoire

**Keywords:** HIV, antiretroviral therapy, sex, attrition, Immunological response, West Africa

## Abstract

**Introduction:**

Sex differences have already been reported in sub‐Saharan Africa for attrition and immunological response after antiretroviral therapy (ART) initiation, but follow‐up was usually limited to the first two to three years after ART initiation. We evaluated sex differences on the same outcomes in the 10 years following ART initiation in West African adults.

**Methods:**

We used cohort data of patients included in the IeDEA West Africa collaboration, who initiated ART between 2002 and 2014. We modelled no‐follow‐up and 10‐year attrition risks, and immunological response by sex using logistic regression analysis, survival analysis with random effect and linear mixed models respectively.

**Results:**

A total of 71,283 patients (65.8% women) contributed to 310,007 person‐years of follow‐up in 16 clinics in eight West African countries. The cumulative attrition incidence at 10‐year after ART initiation reached 75% and 68% for men and women respectively. Being male was associated with an increased risk of no follow‐up after starting ART (5.1% vs. 4.0%, adjusted Odds Ratio: 1.25 [95% CI: 1.15 to 1.35]) and of 10‐year attrition throughout the 10‐year period following ART initiation: adjusted Hazard Ratios were 1.22 [95% CI: 1.17 to 1.27], 1.08 [95% CI: 1.04 to 1.12] and 1.04 [95% CI: 1.01 to 1.08] during year 1, years 2 to 4 and 5 to 10 respectively. A better immunological response was achieved by women than men: monthly CD4 gain was 30.2 and 28.3 cells/mL in the first four months and 2.6 and 1.9 cells/μL thereafter. Ultimately, women reached the average threshold of 500 CD4 cells/μL in their sixth year of follow‐up, whereas men failed to reach it even at the end of the 10‐year follow‐up period. The proportion of patients reaching the threshold was much higher in women than in men after 10 years since ART initiation (65% vs. 44%).

**Conclusions:**

In West Africa, attrition is unacceptably high in both sexes. Men are more vulnerable than women on both attrition and immunological response to ART in the 10 years following ART initiation. Innovative tracing strategies that are sex‐adapted are needed for patients in care to monitor attrition, detect early high‐risk groups so that they can stay in care with a durably controlled infection.

## 1 INTRODUCTION

In 2016, the World Health Organization recommended that all HIV‐infected patients be treated with antiretroviral therapy (ART) irrespective of CD4‐cell counts and clinical stage [1]. The introduction of this universal treatment policy has been an important and positive milestone in the fight against HIV/AIDS. This, however, led to an increased number of patients eligible for ART, thus challenging the delivery of adequate HIV care to both newly eligible patients and those already on ART in resource‐limited settings.

ART improves survival and quality of life in HIV‐positive patients, but only when they remain permanently in care and adhere to treatment [2]. As the number of people living with HIV on ART increases, concerns have been raised for long‐term retention and response to treatment. Several studies have shown that retention of patients on ART was suboptimal across different settings [3, 4, 5, 6, 7, 8, 9]. A systematic review on the data from 13 sub‐Saharan African countries showed 75% and 62% retention rates at 12 and 24 months respectively [9]. Similarly, a meta‐analysis on HIV‐positive adults in 42 low‐and middle‐income countries reported retention rates of 78%, 71% and 69% at 12, 24 and 36 months respectively [4]. More recently, the International epidemiology Databases to Evaluate AIDS (IeDEA) group reported that only 52% of African patients were retained in care five years following ART initiation. This rate improved to 66% after accounting for undocumented mortality and self‐transfers [6].

Factors behind poor retention have been extensively investigated [8, 9, 10, 11, 12, 13, 14, 15, 16], and body of evidence has shown that the reasons behind the loss of patients were generally multi‐factorial. Sex was identified, however, as one of the most consistent determinants for loss to care, with male patients showing excess mortality [17] and higher risk of attrition [8, 14, 18].

Retention in care is the first‐level indicator to estimate ART programme’s effectiveness [2, 19, 20, 21] and to evaluate progress towards the United Nations AIDS Programme (UNAIDS) 90‐90‐90 and 95‐95‐95 targets [22, 23]. However, being retained in care does not guarantee adequate treatment response. In sub‐Saharan Africa, CD4 count is the most commonly available biological marker allowing evaluation of treatment response to ART. Previous studies have reported gender differences in immunological response with better immune reconstitution for women than for men [24, 25, 26].

Retention in care and immunological response in ART‐treated patients have been reported already in many African settings, but less evidence is available from West Africa where the burden of HIV is somewhat lower than in other African regions but where the coverage of care and ART programmes is also far less advanced [27]. In addition, the majority of the available reports assessed the programmatic and clinical outcomes at two to three years following ART initiation only. A previous study on attrition in West Africa did not go further than 12 months of follow‐up on ART [3]. Little is therefore known about how HIV care programmes retain patients in care and provide adequate services in a longer time span and whether the sex difference in attrition persists. We evaluated 10‐year attrition and immunological response to ART by sex among HIV‐infected adults followed in large HIV care programmes throughout West Africa.

## 2 METHODS

### 2.1 The IeDEA West Africa collaboration

The IeDEA international research consortium was established in 2006 by the National Institute of Allergy and Infectious Diseases (NIAID) to consolidate, curate and analyse data on care and treatment of HIV to evaluate the outcomes of people living with HIV/AIDS. IeDEA collects such data from seven international regional data centres, including four in Africa, and one each in the Asia‐Pacific region, the Central/South America/Caribbean region and North America [28].

We conducted a cohort analysis on the data extracted from the IeDEA West Africa collaboration [29]. The present data set included medical records from 16 HIV‐care centres over eight countries participating in this network throughout West Africa: Benin (n = 1), Burkina Faso (n = 2), Côte d’Ivoire (n = 6), Guinee Conakry (n = 1), Mali (n = 2), Nigeria (n = 2), Senegal (n = 1) and Togo (n = 1). Demographic, clinical, biological and therapeutic information was routinely and prospectively collected at these HIV care centres using a standardized data format and submitted to the IeDEA West Africa data coordinating centre based in Côte d’Ivoire.

Data were collected between January 1, 2002 and December 31, 2016 in all clinics and extended to December 31, 2018 at seven clinics in five countries (Benin, Burkina Faso, Côte d’Ivoire (n = 3), Senegal, Togo).

Eligible patients for this study were HIV‐positive adults aged 16 years or older at ART initiation and having started triple combination‐based ART between January 1, 2002 and December 31, 2014. Patients with missing information on either sex, date of birth or date of ART initiation were excluded from the analyses. We considered as CD4 count at baseline the measure is taken at the closest date to ART initiation within a window period of six months prior to and one month after ART initiation. CD4 cell count measurement was recommended every six months in all settings but the actual observed frequency varied across HIV‐care centres.

### 2.2 Statistical analysis

Baseline characteristics of the study population were described by median and frequency and compared by sex using Wilcoxon rank test for quantitative variables and Fisher’s exact test for qualitative variables. The overall probability of attrition at 120 months after ART initiation was described by cumulative incidence functions of different types of drop‐outs (not returning into care after the first visit, death, loss to follow‐up (LTFU) and transfer) and compared by sex.

We distinguished two types of attrition to investigate whether it was influenced by sex. First, we defined patients who never returned into care after ART initiation as “No follow‐up.” Second, patients who died or were lost to follow‐up, that is not returning to the HIV‐care centre for six months or more after being engaged into care, were defined as “Loss to care.” We used logistic regression models to assess the association between sex and the risk of no follow‐up. We then used cause‐specific hazard models to assess the association between sex and loss to care. In this later analysis, patients were right‐censored at either the date of death, the date of transfer, the last date for which they were known to be alive when patients were not seen at the clinic for at least six months without transfer or death report, the date of database end‐point, or 120 months since ART initiation, whichever occurred first. Patients who died but had more than six months of absence from care prior to death were considered as lost to follow‐up and were right‐censored at the last date for which they were known to be alive. Transfer of patient to another clinic was treated as a competing risk. The overall follow‐up time was divided into three periods [Year 1 (M0‐M12), Years 2 to 4 (M13‐M24) and Years 5 to 10 (M25‐M120) following ART initiation], taking into account proportional hazard assumptions. The sample size for each of these three time periods was different since it depended on the number of patients remaining in care at the beginning of the respective period.

In the 10‐year following ART initiation, we also investigated the association between sex and immunological response, defined by the evolution of CD4 count. For this purpose, we included all participants with at least one CD4 count measurement during the follow‐up. We estimated CD4 change with a mixed linear model (MLM) with random intercept and random effect on a two‐phase slope (e.g. fixed effect on CD4 change). Slope phases were first modelled using MLM without predictor, and slope change timing was then determined by comparing models with different timing of the change. We retained the best model according to the Akaike Information Criterion. End‐point definitions were the same as in the previous survival analysis. Sex and other covariates alongside their interaction with time were modelled. Gain in CD4 count over time after ART initiation was compared by sex, and the differences were tested using Wald tests. The estimated absolute CD4 T‐cell/µL change over time was then plotted by sex. Residual homoscedasticity and normality were checked graphically.

Sex was the main explanatory variable for both models on attrition and immunological response. Other covariates in these models were age at baseline (<30, 30 to 40 or >40 years), ART starting period (2002 to 2006, 2007 to 2010 or 2011 to 2014). The logistic regression model was adjusted on CD4 count at baseline and the cause‐specific hazard models were adjusted on CD4 count as a time depending variable. To take into account unobserved heterogeneity between clinical centres, we adjusted all models for a clinic effect by including the study site as a random effect. Clinical stage and body mass index were described at baseline but not included in the models due to the high frequency of missing values.

All statistical analyses were performed using SAS software (version 9.4 for Windows, Copyright (c) 2002 to 2010 by SAS Institute Inc., Cary, NC, USA.)

### 2.3 Ethical approval

The IeDEA West Africa Collaboration obtained authorization from the Ethics committee “Comité de Protection des Personnes Sud‐Ouest et Outre‐mer III” in Bordeaux, France to collect, merge and analyse deidentified data from involved HIV clinics in West Africa. Moreover, each West African site participating in the study obtained authorization from its National Ethics committee.

## 3 RESULTS

### 3.1 Study population characteristics

A total of 73,243 patients were registered in the IeDEA West Africa database. Of these, 1960 were not included due to missing information on sex (n = 4) or baseline therapeutic regimen documented as monotherapy or bi‐therapy (n = 1956). The records of 71,283 patients who initiated triple‐based ART between 2002 and 2014 at one of the IeDEA West Africa HIV care centres were included in the analyses.

Table 1 shows baseline characteristics of the study sample according to sex. Two‐thirds of patients were women (46,893/71,283). Men were significantly older than women at ART initiation with median ages of 40.8 years (Inter Quartile Range [IQR]: 35.1 to 47.2) and 34.0 years (IQR: 29.0 to 40.6) respectively. Overall, CD4 count at baseline was low, but statistically significantly higher in women (184 cells/µL (IQR: 86 to 301)) than in men (140 cells/µL (IQR: 55 to 250)). A substantial proportion of patients had missing CD4 count at baseline (29.8% in men and 28.2 in women). More than half of both male and female patients were started on ART between 2006 and 2010 (55.9% and 56.2% in men and women respectively).

**Table 1:** Characteristics of HIV‐positive adults initiating antiretroviral treatment in HIV clinics participating to the IeDEA West Africa collaboration 2002 to 2018 (N = 71,283)

	Men (N = 24,390)		Women (N = 46,893)		*p*‐value
	n	%	n	%	
Median age at baseline (year) (IQR)	40.8 (35.1 to 47.2)		34.1 (28.9 to 40.7)		<0.001^a^
Age at Baseline (years)					
16 to 30	2194	9.0	14140	30.2	<0.001^b^
30 to 40	9107	37.3	19930	42.5	
>40	13089	53.7	12823	27.3	
Median CD4 cell count (Cells/µL) (IQR)	140 (55 to 250)		184 (86 to 301)		<0.001^a^
Baseline CD4 cell count (Cells/µL)					
<100	6566	38.3	9449	28.1	<0.001^b^
100 to 199	4536	26.5	8547	25.4	
200 to 349	3988	23.3	9454	28.1	
350 to 499	1215	7.1	3295	9.8	
≥500	817	4.8	2913	8.7	
Unknown (%)	7268 (29.8)		13235 (28.2)		
Year of ART initiation					
Prior to 2006	6064	24.9	10873	23.2	0.0143^b^
2006 to 2010	13630	55.9	26340	56.2	
2011 to 2014	4696	19.3	9680	20.6	
Initial ART regimen					
2 NRTIs +1 PI	2625	10.8	4477	9.5	<0.001^b^
2 NRTIs + EFV	11219	46.0	15477	33.0	
2 NRTIs + NVP	9914	40.6	25898	55.2	
Other	632	2.6	1041	2.2	
Body mass index					
Low weight	2362	27.5	5628	31.5	<0.001^b^
Normal	5053	58.7	9272	51.9	
Overweight	1189	13.8	2981	16.7	
Unknown (%)	15786 (64.7)		29012 (61.9)		
Baseline clinical stage					
CDC A/B. WHO I/II	2669	49.6	6302	55.9	<0.001^b^
CDC C. WHO III/ IV	2711	50.4	4970	44.1	
Unknown (%)	19010 (77.9)		35621 (76.0)		

^a^ *p*‐value from Wilcoxon rank sum test.

^b^ *p*‐value from Fisher’s exact test.

### 3.2 Ten‐year risk of attrition since ART initiation by sex

**Figure 1:**
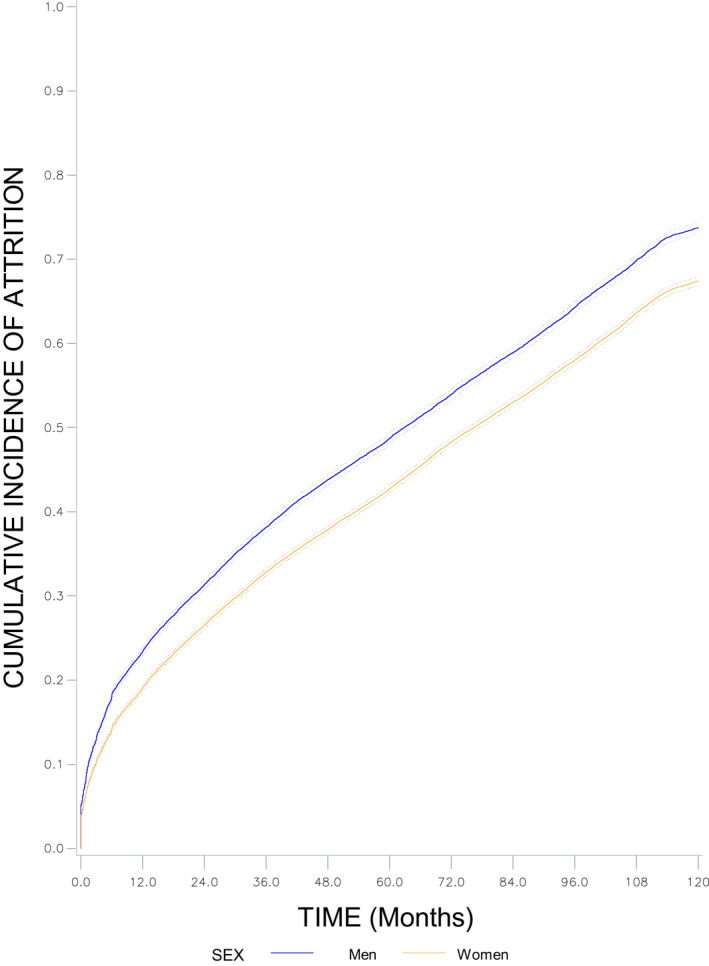
Ten‐year cumulative incidence function of attrition and 95% CI by sex. IeDEA West Africa Collaboration, 2002 to 2018.

Figure 1 represents the cumulative incidence of attrition by sex. Men consistently had a higher probability of attrition compared to women. The probabilities of attrition were 24% and 19% at 12 months, 49% and 43% at 60 months and 74% and 67% at 120 months, for men and women respectively.

**Figure 2:**
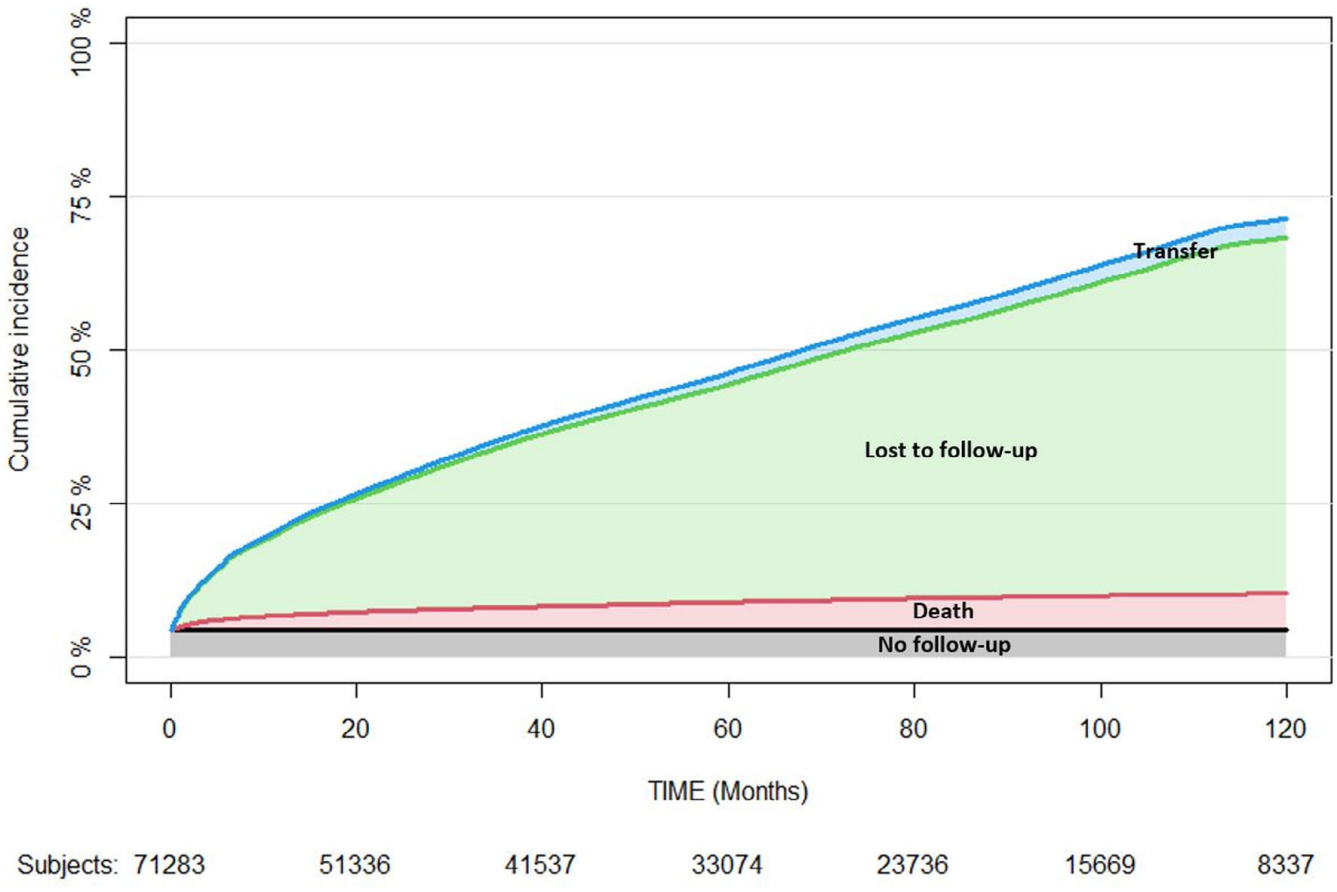
Ten‐year stacked plot of cumulative incidence function of attrition by attrition types. IeDEA West Africa Collaboration, 2002 to 2018.

Figure 2 shows the stacked plot of cumulative incidence of attrition by attrition type (No follow‐up, loss to follow‐up, death, transfer). Overall attrition was 21%, 45% and 71% at 12, 60 and 120 months following ART initiation respectively. Overall, patients lost to follow‐up accounted for 85% of patient’s loss to care. We presented the same figure by sex in Figure S1.

Table 2 shows predictors of no follow‐up visit and attrition during Year 1, Years 2 to 4 and Years 5 to 10 following ART initiation. Men were at a higher risk of never returning into care after ART initiation (5.1% in men vs. 4.0% in women, adjusted Odds Ratios [ORa]: 1.25 [95% CI: 1.15 to 1.35]). Throughout the 10‐year period following ART initiation, men had an increased risk of attrition compared to women: the adjusted Hazard Ratios (HRa) were 1.22 during Year 1 (95% CI: 1.17 to 1.27; 22.6/100 PY in men and 17.5/100 PY in women), 1.08 during Years 2 to 4 (95% CI: 1.04 to 1.12; 10.3/100 PY in men and 8.9/100 PY in women) and 1.05 during Years 5 to 10 (95% CI: 1.01 to 1.08; 11.9/100 PY in men and 10.3/100 PY in women). Having less than 30 years at ART initiation was associated with a higher risk of no‐follow‐up as well as a higher risk of attrition during the follow‐up. Patients who started on ART between 2006 and 2010 or between 2011 and 2014 were significantly more likely to be at‐risk of attrition when compared to those who started on treatment before 2006. This was true for each of the three calendar periods following ART initiation. Low baseline CD4 count (<100 Cells/µL) was associated with no follow‐up and time‐depending CD4 count values were associated with attrition over calendar time, with the risk of attrition decreasing when CD4 increased.

**Table 2:** Predictors of no follow‐up visit and attrition during Year 1, Years 2 to 4 and Years 5 to 10 following ART initiation among HIV‐positive adults in West Africa. IeDEA West‐Africa collaboration, 2002 to 2018

	No follow‐up (n = 71,283)				Year 1 (n = 56,811 PY = 50,375)				Years 2 to 4 (n = 51,985 PY = 129,348)				Years 5 to 10 (n = 37,204 PY = 130,284)				
	n	%	ORa [IC 95%]	*p*‐value	PY	IR/100 PY	HRa [IC 95%]	*p*‐value	PY	IR/100 PY	HRa [IC 95%]	*p*‐value		PY	IR/100 PY	HRa [IC 95%]	*p*‐value
Sex				<0.001				<0.001				<0.001					0.016
Men	24390	5.1	1.25 [1.15; 1.35]		16498	22.6	1.22 [1.17; 1.27]		41930	10.3	1.08[1.04; 1.12]			41621	11.9	1.05[1.01; 1.08]	
Women	46893	4.0	1		33877	17.5	1		87418	08.9	1			88663	10.3	1	
Age at baseline				<0.020				<0.001				<0.001					0.001
<30 years	16334	4.0	1		11497	20.1	1		29141	10.7	1			29517	12.7	1	
30 to 40 years	29037	4.2	0.88 [0.79; 0.97]		20745	18.3	0.87 [0.83; 092]		53689	08.9	0.84 [0.80; 0.87]			55297	10.4	0.90 [0.86; 0.94]	
>40 years	25912	4.8	0.87 [0.78; 0.96]		18133	19.6	0.90[0.85; 0.95]		46518	09.1	0.83 [0.79; 0.87]			45470	10.1	0.93 [0.89; 0.97]	
Year of ART initiation				<0.001				<0.001				<0.001					<0.001
Prior to 2006	16937	3.7	1		10896	16.4	1		32059	07.6	1			51484	07.5	1	
2006 to 2010	39970	4.2	1.51 [1.27; 1.79]		29114	17.9	1.10 [1.04; 1.16]		79803	08.4	1.08 [1.03; 1.14]			74682	13.0	2.34 [2.25; 2.44]	
2011 to 2014	14376	5.7	2.23 [1.85; 2.68]		10365	25.7	1.61 [1.52; 1.72]		17485	17.1	2.65 [2.51; 2.80]			4118	13.5	7.22 [6.53; 7.98]	
Baseline CD4 cell count (Cells/µL)				<0.001													
<100	16015	4.0	1														
100 to 199	13083	2.5	0.61 [0.53; 0.70]														
200 to 349	13442	2.3	0.52 [0.46; 0.60]														
350 to 500	4510	2.8	0.59 [0.48; 0.71]														
≥500	3730	4.0	0.80 [0.66; 0.96]														
Missing	20503	7.7	2.27 [2.05; 2.50]														
Time depending CD4 cell count (Cells/µL)								<0.001				<0.001					<0.001
<100					7283	53.1	1		9820	24.6	1			6134	11.1	1	
100 to 199					10576	21.1	0.56[0.54; 0.59]		14598	15.5	0.53 [0.50; 0.56]			8519	11.1	0.55 [0.52; 0.59]	
200 to 349					15370	13.6	0.44 [0.41; 0.46]		32131	09.6	0.36 [0.35; 0.38]			21309	10.5	0.35 [0.33; 0.37]	
350 to 500					9205	8.5	0.37 [0.34; 0.40]		30896	06.8	0.31 [0.29; 0.33]			28276	12.3	0.31 [0.29; 0.33]	
≥500					7940	8.6	0.38 [0.35; 0.41]		41903	05.4	0.29 [0.27; 0.31]			66046	14.0	0.31 [0.29; 0.32]	

HRa, adjusted cause‐specific Hazard Ratio estimated by Cox regression models; IR/100 PY, incidence rate per 100 person‐years; ORa, adjusted Odd Ratio estimated by the logistic regression model.

### 3.3 Ten‐year immunological response by sex

**Figure 3:**
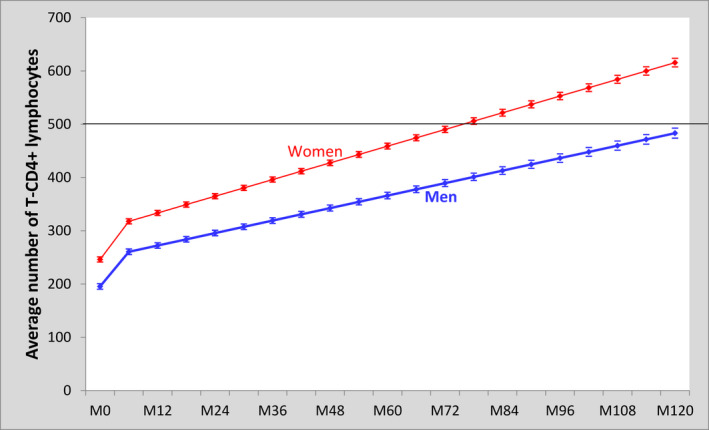
Linear mixed model on the predictive evolution in the average number of CD4 count (95% CI) among women and men in the 10 years following ART initiation. IeDEA West Africa Collaboration, 2002 to 2018.

At ART initiation, the median CD4 count was as low as 184 (86 to 301) and 140 (55 to 250) cells/µL among women and men respectively. The median number of available CD4 measurements all over the 10‐year period was 5 per patient (IQR: 3 to 11). Figure 3 shows the average gain in the number of CD4 cells during the 10 years following ART initiation by sex. A steady increase in the first four months following ART initiation was observed; with an average monthly CD4 gain of 28.3 and 30.2 cells/μL in men and women (*p* < 0.001) respectively. Thereafter, this average monthly CD4 gain was much lower: 1.9 and 2.6 cells/μL in men and women (*p* < 0.001) respectively.

Consequently, the evolution in the average number of CD4 cells was more pronounced in women than in men (Figure 3). The average CD4 count in women reached the 500 CD4 cells/μL threshold in their sisth year of follow‐up while that in men barely reached this threshold at the end of the 10‐year follow‐up period. Of the patients who were retained in care after 10 years since ART initiation, 65% and 44% reached the threshold of 500 CD4 cells/μL in women and men respectively.

We run three separate sensitivity analyses. The first included baseline CD4 count as a confounder in the model and showed similar estimates with persisting sex difference (data not shown). The second looked at patients who remained in care at 10 years and showed similar estimates with persisting sex difference (Figure S2). The third looked at patients with the same baseline CD4 range and showed persisting sex differences for all CD4 ranges (<100; 100 to 200; 200 to 350; >350) (Figure S3).

## 4 DISCUSSION

To the best of our knowledge, this is the first study to provide long‐term follow‐up data aimed at addressing both attrition and immunological response by sex among HIV‐positive adults on ART in West Africa. This analysis was performed on a very large sample of 71.283 adult patients, covering 16 HIV‐care centres across eight countries over a 10‐year period.

Our study confirms the findings of previous studies that men had a higher risk of attrition when compared to women and suggests that this increased risk in men is likely to persist in the long‐term [15, 30, 31]. A study from the IeDEA Southern Africa collaboration showed an excess risk of mortality and LTFU in men during the first 36 months following ART initiation when compared to women [30]. Likewise, studies in Zimbabwe and the Democratic Republic of Congo [8, 18] found a higher risk of attrition in men in the first 12 and 36 months following ART initiation when compared with women. In 2014, a study from three East African countries (Tanzania, Uganda and Zambia) reported an increased risk of attrition among men, however, such sex difference was not found when the health facility benefited from a community‐based approach [14]. In a systematic review published in 2018 including 30 reports from both developed and developing countries, sex was associated with retention in HIV care: men had an increased risk of attrition in resource‐constrained settings while this risk was higher in women in more developed countries [11]. These results highlight the potential role of sex on care‐seeking behaviours, which are different from one place to one another and depend on socio‐cultural and environmental factors. This is the primary reason why differentiated care strategies might offer certain benefits to men’s degree of engagement in care [32].

Our results have also shown that the immunological response was better in women compared to men in the 10 years following ART initiation, regardless of their baseline CD4 count. Women reached the 500 CD4 threshold in their sisth year on ART, whereas men did not reach this threshold after the completion of the 10‐year follow‐up. Our findings are in line with the results of previous studies that showed a better immunological response in women compared to men [33]. In sub‐Saharan Africa, only a few studies investigated the association between sex and CD4 count evolution over time. Available data generally suggest better immunological response in women than men, although the magnitude of the CD4 gain varied across studies [25, 30]. A study from the IeDEA Southern Africa collaboration found a better immunological response in women than men [30] and another large‐scale study incorporating 27 cohorts throughout Africa found no difference between men and women [34]. Such heterogeneity suggests that sex‐specific assumptions should be taken into account in a more specific way, specifically the behavioural differences between women and men in relation to access and use of HIV care services.

Men’s increased risk of attrition and poorer immunological response might be partly explained by the fact that men usually initiate ART at a more advanced stage compared to women [30, 35]. Timely ART initiation in women is related to pregnancy, allowing them to start treatment through the prevention of mother‐to‐child transmission of HIV programmes. On the other hand, men usually initiate ART through standard counselling and testing services [36, 37]. Studies also hypothesized that perceptions of masculinity could play a role in discouraging men to stay in care. Cultural belief that men are independent and invulnerable, even in the event of a disease, may constitute barriers to their engagement into care and ultimately motivate an early exit from HIV care [38, 39, 40, 41, 42, 43].

The extremely high cumulative attrition incidence is worth mentioning, reaching 71% at 10 years after ART initiation, with LTFU contributing to the largest number of attrition cases. This was also observed in Malawi with a retention rate as low as 48% after eight years of follow‐up [7]. These LTFU patients might be either dead or too severely ill to attend health facilities, but they could as well be silent transfers. This raises the issue of traceability of patients in HIV care. A recent meta‐analysis carried out in East, Southern and Central Africa, estimated that 15% of patients classified as LTFU were actually self‐transfers [44]. Another systematic review showed that silent transfers could account for up to 24% of LTFU cases when tracing efforts were successfully put in place [45]. In this systematic review, the authors also reported that the proportion of silent transfers increased over time. The transfer might have occurred due to heavy patient load or insufficient workforce at the health facility to efficiently track patients. Our attrition rate of 71% might has been overestimated since no strategy was in place to trace LTFU patients and to check whether they had been transferred to another HIV care facility or not. Moreover, a number of unreported deaths might have been classified as LTFU. A recent meta‐analysis undertaken in Africa found that 21.8% of patients were confirmed to be dead four years after the last clinical visit [44]. Mortality among LTFU patients was known to be high in the first months and tended to decrease over time whereas undocumented transfers and treatment interruptions tended to increase [45]. We can thus reasonably assume that the mortality rate in our study was probably underestimated, however, the magnitude of such underestimation is difficult to quantify given the context of West Africa where there is no effective vital registry to ascertain death.

Our study has the following limitations. HIV care facilities selected to participate in the IeDEA West Africa collaboration were mostly located in densely populated urban areas and were therefore not representative of all facilities in this region. Limitations also include issues surrounding the completeness, harmonization and management of collected data. These limitations are inherent to this type of cohort analysis, bringing together independent sites with site‐specific modes of operation. The lack of variables characterizing HIV care facilities included in the study that would have allowed us to better adjust our regression models was partly taken into account by the integration of a random component on the site. A large number of missing values in baseline CD4 count was a major challenge, which was overcome to a certain extent by sensitivity analyses focusing on the group of patients with baseline CD4 count. Treatment interruptions were not taken into account in this study, although they are important to understand in order to achieve successful management of programmatic care. Immunological response measured in our study through the estimation of CD4 gain was important for evaluating biological response but incomplete. It is now necessary to apply more direct biological monitoring of ART success by routinely measuring the HIV‐RNA viral load so that the proportion of patients on ART achieving an undetectable HIV‐RNA viral load can be adequately assessed. This requires the strengthening of local laboratory capacities in the West African context largely underserved by international and national HIV programmes.

## 5 CONCLUSIONS

Our study highlights men’s vulnerability to attrition and poorer immunological response to ART in the 10 years following ART initiation. Understanding the reasons behind such a persistent sex difference is important to inform HIV policies and programmes at local level in West Africa in order to achieve the UNAIDS 95‐95‐95 targets by 2030. Bearing in mind the extremely high cumulative attrition incidence among both women and men in the first 10‐year following ART initiation, innovative tracing strategies for LTFU patients are needed for both sexes to better interpret such high attrition rates and to support patients so that they can stay in care in a more sustainable way.

## COMPETING INTEREST

The authors have no conflict of interests to declare.

## AUTHORS’ CONTRIBUTIONS

RB and TT developed the study concept. TT and EB were in charge of the data management. TT performed the statistical analysis. TT, SA and RB interpreted the analyses. TT wrote the first draft of the manuscript with significant inputs from SA and RB. RB supervised the analysis, interpretation and writing process and was primarily responsible for the final content of the manuscript. EM, DE, AT, VK, EB, MDZ, AP, FD, AJ and AM reviewed, provided critical inputs and approved the final manuscript.

## FUNDING

Thierry Tiendrebeogo was a PhD fellow of the French National Research Agency on AIDS and viral hepatitis (ANRS, Grant ANRS 12360 B92). The study was conducted within the International epidemiology Database to Evaluate AIDS (IeDEA) West Africa collaboration grants. It was mainly co‐funded by the National Cancer Institute (NCI), the Eunice Kennedy Shriver National Institute of Child Health & Human Development (NICHD) and the National Institute of Allergy and Infectious Diseases (NIAID) (Grant # 5U01AI069919, Primary investigator: François Dabis). The content is solely the responsibility of the authors and does not necessarily represent the official views of the National Institutes of Health. This study received additional funding from the French charity SIDACTION, Paris, France (Aide aux équipes 15‐2‐AEQ‐06‐01).

## Supporting information


**Figure S1**. 10‐year stacked plot of cumulative incidence function of attrition by attrition types in men and women. IeDEA West Africa Collaboration, 2002 to 2018.
**Figure S2**. Linear mixed model on the predictive evolution in the average number of CD4 count among women and men retained in care in the 10 years following ART initiation. IeDEA West Africa Collaboration, 2002 to 2018.
**Figure S3**. Linear mixed model on the predictive evolution in the average number of CD4 count among women and men by baseline CD4 count category in the 10 years following ART initiation. IeDEA West Africa Collaboration, 2002 to 2018.

## References

[1] WHO . Consolidated guidelines on the use of antiretroviral drugs for treating and preventing HIV infection. Recommendations for a public health approach. Geneva: WHO; 2016 [cited 2020 Jan 2]. Available from: https://www.who.int/hiv/pub/arv/arv‐2016/en/ 27466667

[2] Giordano TP , Gifford AL , White AC Jr , Suarez‐Almazor ME , Rabeneck L , Hartman C , et al. Retention in care: a challenge to survival with HIV infection. Clin Infect Dis. 2007;44(11):1493–9. 17479948 10.1086/516778

[3] Ekouevi DK , Balestre E , Ba‐Gomis FO , Eholie SP , Maiga M , Amani‐Bosse C , et al. Low retention of HIV‐infected patients on antiretroviral therapy in 11 clinical centres in West Africa. Trop Med Int Health. 2010;15:34–42. 20586958 10.1111/j.1365-3156.2010.02505.x PMC2919326

[4] Fox MP , Rosen S . Patient retention in antiretroviral therapy programs up to three years on treatment in sub‐Saharan Africa, 2007–2009: systematic review. Trop Med Int Health. 2010;2009:1–15. 10.1111/j.1365-3156.2010.02508.x PMC2948795 20586956

[5] Fox MP , Rosen S . Retention of adult patients on antiretroviral therapy in low‐ and middle‐income countries: systematic review and meta‐analysis 2008–2013. J Acquir Immune Defic Syndr. 2015;69(1):98–108. 25942461 10.1097/QAI.0000000000000553 PMC4422218

[6] Haas AD , Zaniewski E , Anderegg N , Ford N , Fox MP , Vinikoor M , et al. Retention and mortality on antiretroviral therapy in sub‐Saharan Africa: collaborative analyses of HIV treatment programmes. J Int AIDS Soc. 2018;21:e25084. 10.1002/jia2.25084 PMC5897849 29479867

[7] Mugglin C , Haas AD , van Oosterhout JJ , Msukwa M , Tenthani L , Estill J , et al. Long‐term retention on antiretroviral therapy among infants, children, adolescents and adults in Malawi: a cohort study. PLoS One. 2019;14:e0224837. 31725750 10.1371/journal.pone.0224837 PMC6855432

[8] Mutasa‐Apollo T , Shiraishi RW , Takarinda KC , Dzangare J , Mugurungi O , Murungu J , et al. Patient retention, clinical outcomes and attrition‐associated factors of HIV‐infected patients enrolled in Zimbabwe's National Antiretroviral Therapy Programme, 2007–2010. PLoS One. 2014;9:e86305. 24489714 10.1371/journal.pone.0086305 PMC3906052

[9] Rosen S , Fox MP , Gill CJ . Patient retention in antiretroviral therapy programs in sub‐Saharan Africa: a systematic review. PLoS Med. 2007;4:e298. 17941716 10.1371/journal.pmed.0040298 PMC2020494

[10] Alvarez‐Uria G , Naik PK , Pakam R , Midde M . Factors associated with attrition, mortality, and loss to follow up after antiretroviral therapy initiation: data from an HIV cohort study in India. Glob Health Action. 2013;6:21682. 24028937 10.3402/gha.v6i0.21682 PMC3773168

[11] Bulsara SM , Wainberg ML , Newton‐John TRO . Predictors of adult retention in HIV care: a systematic review. AIDS Behav. 2018;22(3):752–64. 27990582 10.1007/s10461-016-1644-y PMC5476508

[12] Cornell M , Myer L , Kaplan R , Bekker LG , Wood R . The impact of gender and income on survival and retention in a South African antiretroviral therapy programme. Trop Med Int Health. 2009;14(7):722–31. 19413745 10.1111/j.1365-3156.2009.02290.x PMC2771267

[13] Janssen S , Wieten RW , Stolp S , Cremers AL , Rossatanga EG , Klipstein‐Grobusch K , et al. Factors associated with retention to care in an HIV clinic in Gabon, Central Africa. PLoS One. 2015;10:e0140746. 26473965 10.1371/journal.pone.0140746 PMC4608719

[14] Koole O , Tsui S , Wabwire‐Mangen F , Kwesigabo G , Menten J , Mulenga M , et al. Retention and risk factors for attrition among adults in antiretroviral treatment programmes in Tanzania, Uganda and Zambia. Trop Med Int Health. 2014;19(12):1397–410. 25227621 10.1111/tmi.12386 PMC4724698

[15] Takarinda KC , Harries AD , Shiraishi RW , Mutasa‐Apollo T , Abdul‐Quader A , Mugurungi O . Gender‐related differences in outcomes and attrition on antiretroviral treatment among an HIV‐infected patient cohort in Zimbabwe: 2007–2010. Int J Infect Dis. 2015;30:98–105. 25462184 10.1016/j.ijid.2014.11.009 PMC5072602

[16] Tsague L , Koulla SS , Kenfak A , Kouanfack C , Tejiokem M , Abong T , et al. Determinants of retention in care in an antiretroviral therapy (ART) program in urban Cameroon, 2003–2005. Pan Afr Med J. 2008;1:2. 21532891 PMC2984267

[17] Beckham SW , Beyrer C , Luckow P , Doherty M , Negussie EK , Baral SD . Marked sex differences in all‐cause mortality on antiretroviral therapy in low‐ and middle‐income countries: a systematic review and meta‐analysis. J Int AIDS Soc. 2016;19(1):21106. 27834182 10.7448/IAS.19.1.21106 PMC5103676

[18] Koole O , Kalenga L , Kiumbu M , Menten J , Ryder RW , Mukumbi H , et al. Retention in a NGO supported antiretroviral program in the Democratic Republic of Congo. PLoS One. 2012;7:e40971. 22815883 10.1371/journal.pone.0040971 PMC3398868

[19] Braitstein P , Brinkhof MW , Dabis F , Schechter M , Boulle A , Miotti P , et al. Mortality of HIV‐1‐infected patients in the first year of antiretroviral therapy: comparison between low‐income and high‐income countries. Lancet. 2006;367(9513):817–24. 16530575 10.1016/S0140-6736(06)68337-2

[20] Stringer JS , Zulu I , Levy J , Stringer EM , Mwango A , Chi BH , et al. Rapid scale‐up of antiretroviral therapy at primary care sites in Zambia: feasibility and early outcomes. JAMA. 2006;296(7):782–93. 16905784 10.1001/jama.296.7.782

[21] Toure S , Kouadio B , Seyler C , Traore M , Dakoury‐Dogbo N , Duvignac J , et al. Rapid scaling‐up of antiretroviral therapy in 10,000 adults in Côte d'Ivoire: 2‐year outcomes and determinants. AIDS. 2008;22(7):873–82. 18427206 10.1097/QAD.0b013e3282f768f8 PMC3921665

[22] UNAIDS . The Gap Report. Geneva: UNAIDS; 2014 [cited 2020 Jan 2]. Available from: http://files.unaids.org/en/media/unaids/contentassets/documents/unaidspublication/2014/UNAIDS_Gap_report_en.pdf

[23] UNAIDS General Assembly . Resolution No A/RES/70/266, Political Declaration on HIV and AIDS: On the Fast Track to Accelerate the Fight against HIV and to End the AIDS Epidemic by 2030. 2016 [cited 2020 Jan 2]. Available from: https://www.unaids.org/sites/default/files/media_asset/2016‐political‐declaration‐HIV‐AIDS_en.pdf

[24] Balestre E , Eholié SP , Lokossue A , Sow PS , Charurat M , Minga A , et al. Effect of age on immunological response in the first year of antiretroviral therapy in HIV‐1‐infected adults in West Africa. AIDS. 2012;26(8):951–7. 22382142 10.1097/QAD.0b013e3283528ad4 PMC3704338

[25] Maman D , Pujades‐Rodriguez M , Subtil F , Pinoges L , McGuire M , Ecochard R , et al. Gender differences in immune reconstitution: a multicentric cohort analysis in sub‐Saharan Africa. PLoS One. 2012;7:e31078. 22363550 10.1371/journal.pone.0031078 PMC3281917

[26] Sempa JB , Kiragga AN , Castelnuovo B , Kamya MR , Manabe YC . Among patients with sustained viral suppression in a resource‐limited setting, CD4 gains are continuous although gender‐based differences occur. PLoS One. 2013;8:e73190. 24013838 10.1371/journal.pone.0073190 PMC3754935

[27] UNAIDS . Global AIDS Update – Seizing the moment–Tackling entrenched inequalities to end epidemics. Geneva: UNAIDS; 2020 [cited 2020 Jul 9]. Available from: https://www.unaids.org/sites/default/files/media_asset/2020_global‐aids‐report_en.pdf

[28] IeDEA International epidemiology Databases to Evaluate AIDS [Internet]. IeDEA International epidemiology Databases to Evaluate AIDS [cited 2020 Jul 9]. Available from: https://www.iedea.org/

[29] Egger M , Ekouevi DK , Williams C , Lyamuya RE , Mukumbi H , Braitstein P , et al. Cohort Profile: the international epidemiological databases to evaluate AIDS (IeDEA) in sub‐Saharan Africa. Int J Epidemiol. 2012;41(5):1256–64. 21593078 10.1093/ije/dyr080 PMC3465765

[30] Cornell M , Schomaker M , Garone DB , Giddy J , Hoffmann CJ , Lessells R , et al. Gender differences in survival among adult patients starting antiretroviral therapy in South Africa: a multicentre cohort study. PLoS Medicine. 2012;9:e1001304. 22973181 10.1371/journal.pmed.1001304 PMC3433409

[31] Cornell M , Johnson LF , Wood R , Tanser F , Fox MP , Prozesky H , et al. Twelve‐year mortality in adults initiating antiretroviral therapy in South Africa. J Int AIDS Soc. 2017;20:21902. 28953328 10.7448/IAS.20.1.21902 PMC5640314

[32] Hagey JM , Li X , Barr‐Walker J , Penner J , Kadima J , Oyaro P , et al. Differentiated HIV care in sub‐Saharan Africa: a scoping review to inform antiretroviral therapy provision for stable HIV‐infected individuals in Kenya. AIDS Care. 2018;30(12):1477–87. 30037312 10.1080/09540121.2018.1500995

[33] Nash D , Katyal M , Brinkhof MW , Keiser O , May M , Hughes R , et al. Long‐term immunologic response to antiretroviral therapy in low‐income countries: a collaborative analysis of prospective studies. AIDS. 2008;22(17):2291–302. 18981768 10.1097/QAD.0b013e3283121ca9 PMC2794130

[34] Novelli S , Delobel P , Bouchaud O , Avettand‐Fenoel V , Fialaire P , Cabié A , et al. Enhanced immunovirological response in women compared to men after antiretroviral therapy initiation during acute and early HIV‐1 infection: results from a longitudinal study in the French ANRS Primo cohort. J Int AIDS Soc. 2020;23:e25485. 32333726 10.1002/jia2.25485 PMC7183251

[35] Taylor‐Smith K , Tweya H , Harries A , Schoutene E , Jahn A . Gender differences in retention and survival on antiretroviral therapy of HIV‐1 infected adults in Malawi. Malawi Med J. 2010;22(2):49–56. 21614882 10.4314/mmj.v22i2.58794 PMC3345762

[36] Lahuerta M , Lima J , Nuwagaba‐Biribonwoha H , Okamura M , Alvim MF , Fernandes R , et al. Factors associated with late antiretroviral therapy initiation among adults in Mozambique. PLoS One. 2012;7:e37125. 22615917 10.1371/journal.pone.0037125 PMC3352894

[37] Ndawinz JD , Chaix B , Koulla‐Shiro S , Delaporte E , Okouda B , Abanda A , et al. Factors associated with late antiretroviral therapy initiation in Cameroon: a representative multilevel analysis. J Antimicrob Chemother. 2013;68(6):1388–99. 23391713 10.1093/jac/dkt011

[38] Cornell M . Gender inequality: bad for men's health. South Afr J HIV Med. 2013;14(1):12–4. 24078805 10.7196/SAJHIVMED.894 PMC3782744

[39] Fitzgerald M , Collumbien M , Hosegood V . "No one can ask me 'Why do you take that stuff?'": men's experiences of antiretroviral treatment in South Africa. AIDS Care. 2010;22(3):355–60. 20390516 10.1080/09540120903111536

[40] Jacques‐Aviñó C , García de Olalla P , González Antelo A , Fernández Quevedo M , Romaní O , Caylà JA . The theory of masculinity in studies on HIV. A systematic review. Glob Public Health. 2019;14(5):601–20. 29972098 10.1080/17441692.2018.1493133

[41] Nyamhanga TM , Muhondwa EP , Shayo R . Masculine attitudes of superiority deter men from accessing antiretroviral therapy in Dar es Salaam, Tanzania. Glob Health Action. 2013;6:21812. 24152373 10.3402/gha.v6i0.21812 PMC3807014

[42] Siu GE , Seeley J , Wight D . Dividuality, masculine respectability and reputation: how masculinity affects men's uptake of HIV treatment in rural eastern Uganda. Soc Sci Med. 2013;89:45–52. 23726215 10.1016/j.socscimed.2013.04.025

[43] Skovdal M , Campbell C , Madanhire C , Mupambireyi Z , Nyamukapa C , Gregson S . Masculinity as a barrier to men's use of HIV services in Zimbabwe. Global Health. 2011;7:13. 21575149 10.1186/1744-8603-7-13 PMC3107786

[44] Chammartin F , Zürcher K , Keiser O , Weigel R , Chu K , Kiragga AN , et al. Outcomes of patients lost to follow‐up in AFRICAN antiretroviral therapy programs: individual patient data meta‐analysis. Clin Infect Dis. 2018;67(11):1643–52. 29889240 10.1093/cid/ciy347 PMC6233676

[45] Zürcher K , Mooser A , Anderegg N , Tymejczyk O , Couvillon MJ , Nash D , et al. Outcomes of HIV‐positive patients lost to follow‐up in African treatment programmes. Trop Med Int Health. 2017;22(4):375–87. 28102610 10.1111/tmi.12843 PMC5580236

