# Supplementary material for: Ten‐year attrition and antiretroviral therapy response among HIV‐positive adults: a sex‐based cohort analysis from eight West African countries
Source: J Int AIDS Soc. 2021 May 21;24(5):e25723. doi: 10.1002/jia2.25723 (PMC8140184; doi:10.1002/jia2.25723)
Supplement: Supplementary file 1 — Figure S1. 10‐year stacked plot of cumulative incidence function of attrition by attrition types in men and women. IeDEA West Africa Collaboration, 2002 to 2018. Figure S2. Linear mixed model on the predictive evolution in the average number of CD4 count among women and men retained in care in the 10 years following ART initiation. IeDEA West Africa Collaboration, 2002 to 2018. Figure S3. Linear mixed model on the predictive evolution in the average number of CD4 count among women and men by baseline CD4 count category in the 10 years following ART initiation. IeDEA West Africa Collaboration, 2002 to 2018. [file JIA2-24-e25723-s001.docx]

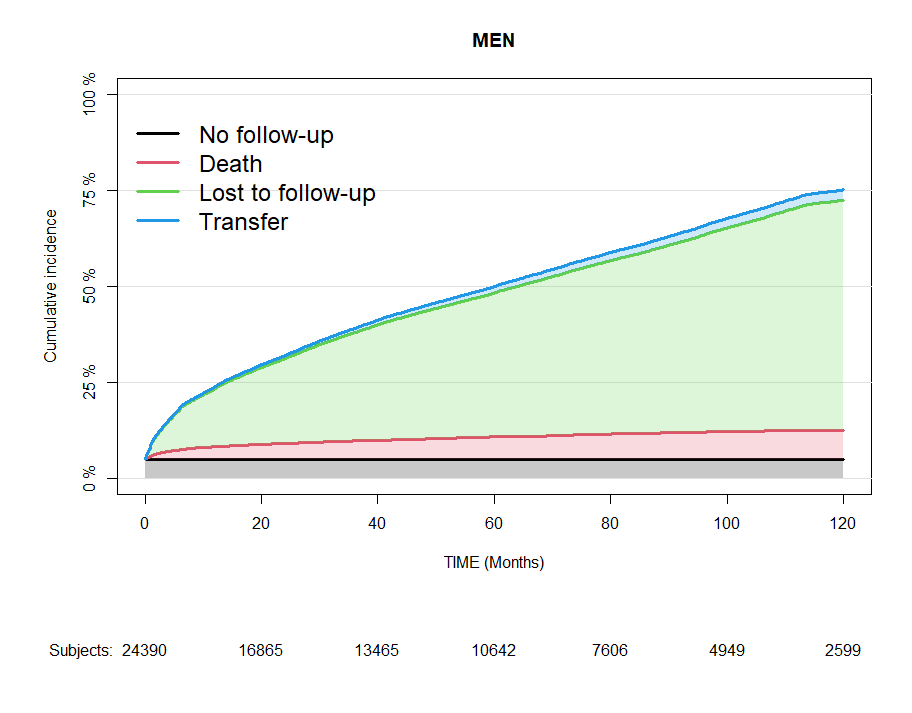


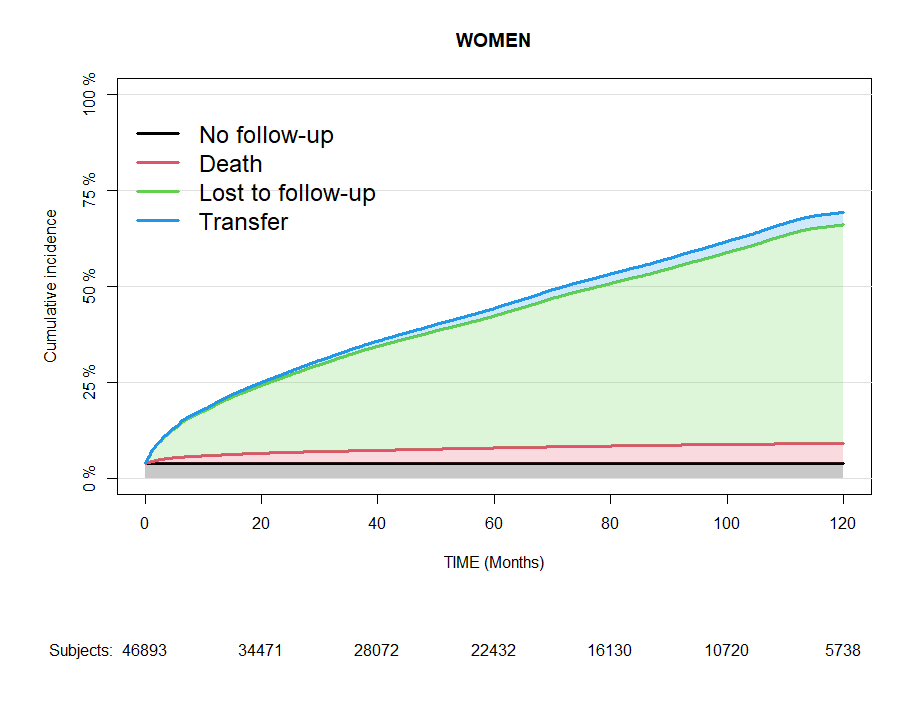


**Supplementary Fig 1.** 10-year stacked plot of cumulative incidence function of attrition by attrition types in men and women. IeDEA West Africa Collaboration, 2002-2018.

**Supplementary Fig 2.** Linear mixed model on the predictive evolution in the average number of CD4 count among women and men retained in care in the 10 years following ART initiation. IeDEA West Africa Collaboration, 2002-2018.

**Baseline CD4 count <100 cells/μl**

**200 cells/μl <=Baseline CD4 count <350 cells/μl**

**100 cells/μl <=Baseline CD4 count <200 cells/μl**

**Baseline CD4 count >=350 cells/μl**

**Supplementary Fig 3.** Linear mixed model on the predictive evolution in the average number of CD4 count among women and men by baseline CD4 count category in the 10 years following ART initiation. IeDEA West Africa Collaboration, 2002-2018.
